# Transition to Advanced Heart Failure: From Identification to Improving Prognosis

**DOI:** 10.3390/jcdd12030104

**Published:** 2025-03-17

**Authors:** Nikolaos-Iason Tepetes, Christos Kourek, Adamantia Papamichail, Andrew Xanthopoulos, Peggy Kostakou, Ioannis Paraskevaidis, Alexandros Briasoulis

**Affiliations:** 1 Department of Clinical Therapeutics, Alexandra Hospital, National and Kapodistrian University of Athens, 11528 Athens, Greece; tepetesnikos@gmail.com (N.-I.T.); pkostakou@gmail.com (P.K.); 2 Department of Cardiology, 417 Army Share Fund Hospital of Athens (NIMTS), 11521 Athens, Greece; chris.kourek.92@gmail.com; 3 Medical School of Athens, National and Kapodistrian University of Athens, 15772 Athens, Greece; adamantiapm@gmail.com; 4 Department of Cardiology, University Hospital of Larissa, 41110 Larissa, Greece; andrewxanth@gmail.com; 5 Department of Cardiology, Hygeia Hospital, 15123 Athens, Greece; iparas@otenet.gr

**Keywords:** advanced heart failure, guideline-directed therapy, therapeutic strategy, palliative care, devices, cardiopulmonary exercise testing

## Abstract

Advanced heart failure (AHF) represents the terminal stage of heart failure (HF), characterized by persistent symptoms and functional limitations despite optimal guideline-directed medical therapy (GDMT). This review explores the clinical definition, pathophysiology, and therapeutic approaches for AHF. Characterized by severe symptoms, New York Heart Association (NYHA) class III-IV, significant cardiac dysfunction, and frequent hospitalizations, AHF presents substantial challenges in prognosis and management. Pathophysiological mechanisms include neurohormonal activation, ventricular remodeling, and systemic inflammation, leading to reduced cardiac output and organ dysfunction. Therapeutic strategies for AHF involve a multidisciplinary approach, including pharmacological treatments, device-based interventions like ventricular assisted devices, and advanced options such as heart transplantation. Despite progress, AHF management faces limitations, including disparities in access to care and the need for personalized approaches. Novel therapies, artificial intelligence, and remote monitoring technologies offer future opportunities to improve outcomes. Palliative care, which focuses on symptom relief and quality of life, remains crucial for patients ineligible for invasive interventions. Early identification and timely intervention are pivotal for enhancing survival and functional outcomes in this vulnerable population. This review underscores the necessity of integrating innovative technologies, personalized medicine, and robust palliative strategies into AHF management to address its high morbidity and mortality.

## 1. Definition of Advanced Heart Failure

### 1.1. Clinical Criteria

Advanced heart failure (AHF) is the stage of heart failure (HF) in which patients continue to have symptoms and functional impairment even after receiving guideline-directed medical therapy (GDMT) [1]. Based on specific clinical criteria, the comprehension of AHF aims to identify patients who may benefit from advanced treatment options such as heart transplantation (HT), device implantation, or palliative care (e.g., inotropes) [2,3].

In order for AHF to be defined, the Heart Failure Society of America Guidelines Committee declares that all of the following criteria must be met even with the most effective treatment according to guidelines: severe and persistent HF symptoms (New York Heart Association, NYHA class III or IV), severe cardiac dysfunction, as indicated by left ventricular ejection fraction (LVEF) ≤ 30%, and hospitalizations or unscheduled visits for HF episodes during the previous 12 months [4]. This classification highlights the significance of identifying patients who may need advanced treatments and are resistant to conventional alternatives to therapy.

Additionally, prompt referral to AHF-specialized facilities is essential for enhancing patient outcomes since it facilitates early therapies that can have a major impact on AHF patients’ survival [5,6].

#### 1.1.1. NYHA Class III-IV Symptoms

**Figure 1:**
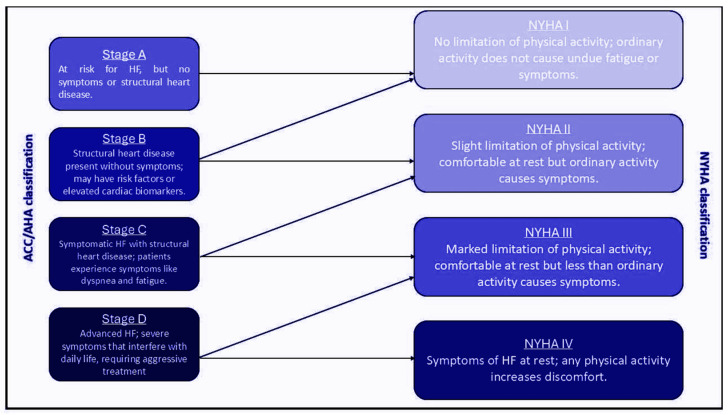
Schematic presentation of HF progression. This figure illustrates diagrammatically the transition of HF from Stage A to Stage D, as classified by the ACC/AHA. Each stage is associated with specific clinical characteristics and symptoms. The NYHA functional classification is also depicted, indicating the patient’s functional capacity at each stage. HF: heart failure, ACC/AHA: American College of Cardiology/American Heart Association, NYHA: New York Heart Association.

The clinical course of patients with HF is unpredictable in the vast majority of cases, as there are many factors that can influence the clinical presentation, such as different responses to treatment or different underlying conditions, both of cardiac and extracardiac origin [6]. The NYHA functional classification is a widely used clinical tool for the stratification of HF severity (Figure 1). Patients with symptoms of severe functional impairment (NYHA class III or IV), who experience symptoms of severe HF, like severe fatigue, dyspnea, or other manifestations even at minimal activity or at rest, often with dependence on intravenous inotropic drugs or repeated hospitalizations, have been classified as AHF patients [7,8]. A percentage of approximately 20–30% of HF patients progress to AHF, a condition characterized by significant limitations in exercise capacity and quality of life [9]. Moreover, AHF stage indicates the presence of severe systolic or diastolic dysfunction, often associated with reduced cardiac output and elevated filling pressures and reduced cardiac output, as compensatory mechanisms (e.g., neurohormonal activation) fail to sustain adequate tissue perfusion and oxygenation. Finally, AHF patients experience a one-year mortality rate exceeding 30%, despite receiving GDMT [10]. This high mortality rate underscores the critical need for effective management strategies and timely intervention to improve patient outcomes in this vulnerable population.

#### 1.1.2. Dependence on Intravenous Inotropic Support or Recurrent Hospitalizations

Patients with AHF may remain dependent on intravenous inotropic drugs (e.g., dobutamine, milrinone) for life or require frequent hospitalizations for acute episodes of instability to preserve their hemodynamic stability.

Inotropic agents are used in patients with low-output HF to help maintain cardiac output and prevent end-organ hypoperfusion. Approximately 5–10% of these patients are dependent on them, reflecting an end-stage disease [11]. However, their use is not without health risks; long-term inotropic treatment is associated with arrhythmias, ischemia, and a significant increase in mortality, with a hazard ratio (HR) exceeding 2. Most HF-related hospitalizations and deaths occur in the subgroup of patients with AHF [12]. Recurrent hospitalizations represent a main feature of AHF. In turn, each recurrent hospitalization predicts treatment failure, disease progression, and worse survival, with the risk of mortality increasing by 20–30% after each event of HF decompensation [10].

The Acute Decompensated Heart Failure National Registry Longitudinal Module (ADHERE LM) revealed that, during a mean follow-up of 364 days, 59% of AHF patients were admitted to the hospital. Among them, 37% were hospitalized one time, 23% two times, 14% three times, 9% four times, and 18% ≥ five times [12,13]. Moreover, the Epidémiologie de l’Insuffisance Cardiaque Avancée en Lorraine (EPICAL) study, conducted on 2577 patients with AHF with a mean follow-up of 18 months, revealed that patients were hospitalized an average of 2.05 times per year, spending a total of 27.6 days per year in the hospital [12,14].

#### 1.1.3. Refractory Symptoms Despite Optimal GDMT

Patients with AHF experience symptoms that are resistant to GDMT due to progressive malfunctioning mechanisms. Recent guidelines from the European Society of Cardiology (ESC) and the American Heart Association (AHA) emphasize the importance of diagnosing AHF using specific criteria. GDMT includes medications such as angiotensin receptor–neprilysin inhibitors (ARNIs), beta-blockers (e.g., carvedilol and bisoprolol), mineralocorticoid receptor antagonists (MRAs), ACE inhibitors (ACEis), and SGLT2 inhibitors (SGLT2is). However, refractory HF is characterized by persistent symptoms, including fluid overload, fatigue, and dyspnea, despite optimal medical therapy. The hemodynamic profile of patients with AHF usually reveals low cardiac index (<2.2 111 L/min/m^2^) and high pulmonary capillary wedge pressure (>18 mmHg), reflecting significant hemodynamic impairment. As the disease progresses, structural and functional deterioration, including extensive fibrosis, myocyte death, and chronic inflammation, further exacerbate cardiac dysfunction. The prognosis for patients with refractory symptoms remains poor, with a one-year survival rate of approximately 50%, underscoring the urgent need for advanced therapeutic strategies and timely interventions to improve outcomes.

### 1.2. Epidemiology

Although evidence-based therapies have led to improved outcomes for patients with chronic HF, challenges remain [5]. In recent years, the incidence of AHF has been increasing, largely due to the prolongation of life expectancy. However, unlike chronic HF, determining the prevalence of AHF remains an epidemiological challenge, as it is influenced by its relatively lower occurrence and the continuous evolution of treatment options. The prevalence of AHF appears to increase with age, primarily affecting older adults (over 65 years), where it reaches up to 10% [10]. Additionally, AHF is more commonly observed in males, likely due to a higher predisposition to ischemic heart disease (Table 1 and Table 2).

In terms of incidence, AHF represents approximately 5–10% of all HF cases worldwide. Within the general population, its estimated incidence is around 23 cases per 1000 individuals per year [15].

The annual mortality for AHF can reach 20–50%, despite modern therapeutic interventions [16].

Common comorbidities that worsen prognosis include diabetes mellitus (40%), chronic kidney disease (30–50%), hypertension (60–70%), and arrhythmias such as atrial fibrillation (AF) [16]. Women with AHF tend to be older and exhibit higher rates of hypertension and diastolic HF compared to their male counterparts [15].

Regarding racial disparities, African Americans are approximately twice as likely to develop HF compared to Caucasians, primarily due to a higher incidence of hypertension and obesity [15]. Hispanic patients often have less access to specialized treatments, contributing to increased mortality rates in this group [16]. In developed countries, the incidence of AHF is rising due to an aging population and improved survival following acute cardiac events, while in developing countries, AHF is more commonly associated with rheumatic heart disease and delayed diagnosis. As a consequence, AHF significantly increases healthcare costs due to frequent hospitalizations and expensive treatments such as implantable defibrillators and cardiac support devices [17]. The quality of life for these patients is often poor, with substantial limitations in daily activities.

A 2023 study showed that the mortality rates for HF were lower in patients aged 65–74 years and women compared to younger patients and men, respectively. Mortality was higher in patients from rural areas and those taking beta-blockers and ACEis/angiotensin receptor blockers/ARNIs. Hospitalization risk was lower in patients aged 65–74 years and 75 years and older compared to patients under the age of 65 years. Hospitalization risk did not differ by gender or rurality. Hospitalization risk was higher in Black patients compared to White patients, while Asian patients were at lower risk. Adjusted all-cause hospitalization risk was modestly but significantly higher for all comorbidities evaluated except for dementia and cerebrovascular disease. Patients aged 65–74 years and women were at lower risk for HF hospitalization compared to younger patients and men, respectively [3].

### 1.3. Pathophysiology

The pathophysiology of AHF is complex and multifactorial (Table 3). AHF is characterized by mechanisms of progressive dysfunction that include neurohormonal activation and ventricular remodeling, changes that can explain why AHF is resistant to traditional treatment options. These are dynamic changes, which affect both the structure and the function of the cardiac muscle.

On the one hand, neurohormonal activation includes the enhancement of the renin–angiotensin–aldosterone axis (RAAS) and sympathetic nervous system (SNS) activity. The first one leads to vasoconstriction, fluid retention, increased afterload, and, thus, the deterioration of hemodynamic function. Moreover, increased aldosterone levels promote fibrosis of the myocardium and worsen diastolic function. Chronic SNS hyperactivity increases afterload and decreases cardiac output via β-adrenergic downregulation [18,19].

On the other hand, drastic left ventricular LV remodeling includes hypertrophy, dilatation and reduction in contractility in response to chronic pressure and volume overload, and increased end-systolic volume. The end-systolic and end-diastolic size of the left ventricle increases, leading to a further decrease in cardiac output. Impaired LV relaxation leads to increased end-diastolic pressures and pulmonary congestion. Chronic pressure and volume overload cause myocardial fibrosis and hypertrophy, which reduce elasticity and increase intracardiac pressure. With regard to imaging features, mid-ventricular obstruction can be seen in AHF patients, which is an ominous sign of increased mortality risk [18,19,20,21,22].

Moreover, inflammation and oxidative stress also play a pivotal role in the development of AHF. Increased levels of pro-inflammatory cytokines, such as tumor necrosis factor (TNF-α) and interleukin 6 (IL-6), promote cardiac dysfunction through the inhibition of contractility and activation of apoptosis. Oxidative stress damages mitochondria and reduces energy production, deteriorating myocardial cell function [18,20,23].

Factors that can lead to AHF include ischemic heart disease, hypertension, and others. Ischemic heart disease remains the leading cause of heart failure. Chronic myocardial ischemia and acute myocardial infarctions lead to the loss of functional myocardium. Chronic hypertension increases afterload, causing LV hypertrophy and, eventually, dysfunction. Valvular diseases can also trigger AHF. Significant stenosis or regurgitation of the mitral and aortic valves causes volume or pressure overload, leading to AHF. Finally, the dysregulation of comorbidities such as renal failure, arrhythmias, or infections can destabilize cardiac function. Additionally, poor adherence to medications or dietary restrictions can exacerbate congestion, further compromising cardiovascular stability [18,20,24]. All the above-mentioned pathophysiological changes can lead to hemodynamic instability and multisystem involvement. Indeed, AHF affects renal function, causes pulmonary hypertension, and increases the likelihood of thromboembolic events.

**Table 3:** Pathophysiological mechanisms underlying AHF.

Mechanism	Description
Neurohormonal activation	Activation of RAAS, SNS [18,19]
Cardiac remodeling	Systolic/diastolic dysfunction and fibrosis [18,21]
Inflammation	Elevation of IL-6 and TNFα levels [18,23]
Oxidative stress	Mitochondrial dysfunction [20,23]

## 2. Transitioning to Advanced Heart Failure

**Figure 2:**
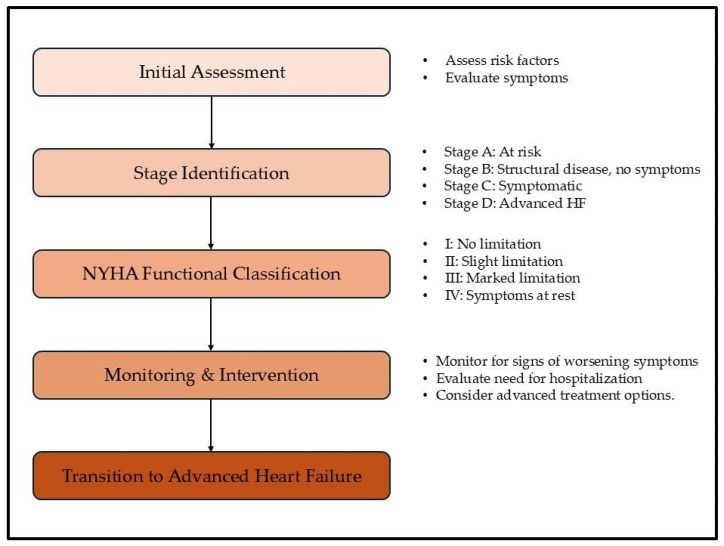
Flowchart for identifying transition to AHF. This flowchart outlines the clinical decision-making process for identifying patients transitioning to AHF. It begins with an initial assessment of risk factors and symptoms, followed by the classification of heart failure stages according to the ACC/AHA Guidelines. The flowchart incorporates the NYHA functional classification to evaluate patients’ functional capacity. Key monitoring and intervention strategies are highlighted to facilitate timely recognition (assessment of risk factors includes obesity, diabetes, and hypertension, and evaluation of symptoms includes dyspnea and fatigue) of AHF, ensuring that appropriate management and treatment options (e.g., LVAD, HT) are considered for patients exhibiting severe symptoms. HF: heart failure, ACC/AHA: American College of Cardiology/American Heart Association, NYHA: New York Heart Association, HT: heart transplantation, LVAD: left ventricular assist device.

The transition from stable chronic heart failure to AHF (Stage D) is characterized by a clinical deterioration of the patient’s symptoms that persist despite the optimization of GDMT (Figure 2) [25].

At this phase, recurrent hospitalizations occur and a need for positive inotropic agents emerges because of persisting symptoms such as congestion, worsening end-organ function, ventricular arrhythmias, and hypoperfusion [26]. These patients are unable to tolerate optimal medical therapy and are classified under NYHA functional classes III or IV (AHA Stage D). They exhibit significantly reduced exercise capacity (peak oxygen consumption VO_2_ < 14 mL/kg/min) and experience severe symptoms, including dyspnea, fatigue, and volume overload. Pulmonary congestion and peripheral edema persist despite high-dose oral diuretics (>160 mg furosemide per day), indicating refractory fluid retention [27]. Weight loss may also be present due to impaired functional and metabolic status, leading to cachexia. Mechanical circulatory support and cardiac transplantation become necessary at this point; otherwise, the median survival is less than 2 years [27]. In many cases, hypotension leads to the intolerance of neurohormonal therapies like angiotensin-converting enzyme (ACE) inhibitors or beta-blockers, further indicating poor prognosis. Worsening chronic HF is often underappreciated when it does not lead to hospitalization, as hospitalization alone cannot fully capture the pathophysiology of the disease [27]. HF progresses both in outpatient settings and during hospital admissions, making it essential to evaluate worsening HF independently of hospitalization status due to its inherently poor prognosis. The importance lies in the prompt identification of the transition to AHF, which will allow for early patient referral to specialized centers that can provide advanced treatments [28].

Early identification of the transition to AHF is crucial for improving patient management and outcomes [29]. By utilizing risk scores, biomarkers, and non-invasive diagnostic tools, experts can evaluate disease progression more efficiently and accurately identify high-risk individuals so that they can intervene in a timely manner to prevent further deterioration. For instance, the I NEED HELP acronym can be used as an evidence-based guide for clinicians in order to suspect the transition to Stage D HF and perhaps seek assistance from specialized HF centers [30]. It stands for inotropic support, NYHA IV functional class, end-organ dysfunction (e.g., liver, kidney), ejection fraction < 20%, defibrillator shocks indicating malignant ventricular arrhythmias, hospitalizations (>1 within the last year), edema (or escalating diuretics), low systolic blood pressure (<90 mmHg), and prognostic medication (inability to up-titrate GDMT). It includes the core clinical characteristics that increase all-cause mortality in patients with AHF and should be a useful tool for a timely referral. High levels of biomarkers like B-type natriuretic peptide (BNP) or N-terminal pro-B-type natriuretic peptide (NT-proBNP) further signal AHF in the absence of non-cardiac causes. However, while BNP is widely used as a biomarker in HF, its predictive value for worsening outcomes was minor in comparison to other clinical factors (age, NYHA class, ejection fraction, etc.) [30]. This suggests that clinical parameters can provide more relevant information for risk stratification in this setting than BNP levels alone. Hyponatremia (sHR = 2.18, *p* < 0.001), a 6 min walk test distance of less than 300 m, and serum sodium levels below 134 mEq/L serve as important warning indicators. The annual progression rates were significantly higher among Black patients compared to White patients (6.3% vs. 2.7%; *p* < 0.001) in individuals with nonischemic versus ischemic heart failure (6.1% vs. 2.9%; *p* < 0.001), and in those with NYHA functional class III to IV symptoms compared to class I to II (7.5% vs. 1.9%; *p* < 0.001). However, no significant difference in progression rates was observed between men and women (4.7% vs. 4.2%; *p* = 0.53) [25].

Approximately 4.5% of patients with Stage C HF progress to end-stage HF each year, highlighting the need for non-invasive assessments like cardiopulmonary exercise testing (CPET). CPET offers a highly accurate evaluation of exercise capacity, providing valuable insight into overall cardiac function [31]. CPET yields several significant parameters which can be used for risk assessment in AHF. Peak VO_2_ is the most objective measure of functional capacity in HF patients, with lower values indicating a poorer prognosis. A peak VO_2_ ≤ 12 mL/kg/min (or ≤50% of predicted VO_2_) has been approved as an eligibility criterion for cardiac transplantation. Peak VO_2_ < 10 mL/kg/min, ventilatory efficiency (VE/VCO_2_) slope ≥ 36, and VO_2_ anaerobic threshold < 11 mL/kg/min suggest poor prognosis and are even associated with >20% mortality within 1 year [32]. De Groote et al. conducted a population-based study aiming to highlight the prognostic value of VO_2_ and BNP levels in patients with congestive HF [33]. Multivariate analysis showed that the percentage achieved of predicted peak oxygen uptake (%VO_2_) had the highest prognostic value for both cardiac survival (RR = 2.84, *p* < 0.0001) and cardiac event-free survival (RR = 2.99, *p* < 0.0001). A %VO_2_ cutoff of 50% could significantly risk stratifying HF patients. In the HF-ACTION trial, a 6% improvement in peak VO_2_ (approximately 1 mL/kg/min) was associated with a 5% reduction in the risk of death or hospitalization [34]. In 1991, Mancini et al. conducted the first study evaluating the prognostic value of peak VO_2_ and the VE/VCO_2_ slope. Their findings supported the use of peak VO_2_ for risk stratification, though the Weber classification system (A, B, C, D) was not originally proposed by them [35]. To accurately assess risk, physicians must determine whether the patient has exerted maximum effort during testing. This is typically indicated by a Respiratory Exchange Ratio (RER) > 1 to 1.1, along with a heart rate exceeding 85% of the predicted value (though β-blocker use may limit this measure). The Weber classification categorizes patients based on peak VO_2_: Class A (>20 mL/kg/min), Class B (16–20 mL/kg/min), Class C (10–16 mL/kg/min), and Class D (<10 mL/kg/min). These classes correspond to 3-year survival rates without the need for transplantation or mechanical circulatory support (MCS) at 97%, 93%, 83%, and 64%, respectively [36]. Nadruz et al. analyzed the results of cardiopulmonary exercise testing (CPET) in a cohort of 969 patients, categorizing them based on their ejection fraction into three groups: (1) preserved ejection fraction (EF > 50%), (2) mid-range ejection fraction (EF 40–49%), and (3) reduced ejection fraction (EF < 40%) [37]. This study showed statistically significant variations among these categories. Peak VO_2_ was found to be lower in HFrEF (14.3 ± 5.2 mL/kg/min) compared to HF with a mildly reduced ejection fraction (HFmrEF) (17.1 ± 7.1 mL/kg/min) and HF with preserved ejection fraction (HFpEF) (17.4 ± 7.8 mL/kg/min). VE/VCO_2_ slope was higher in HF with reduced ejection fraction (HFrEF) (34.5 ± 9.2) than in HFmrEF (29.5 ± 6.3) and HFpEF (30.3 ± 6.7). In addition, HFrEF patients had a lower peak heart rate, as well as lower systolic and diastolic blood pressure, compared to the other two groups. Analysis revealed that both peak VO_2_ and VE/VCO2 slope independently predicted deaths, left ventricular assist device (LVAD) implantation, and heart transplantation [37].

However, complications arise due to the varying classification criteria used in each study. Ho et al. focused on patients with symptomatic HFpEF (NYHA II-IV) and examined their clinical characteristics and CPET results [38]. They utilized the classification systems from the ESC, ACC/AHA, and HFSA to categorize the patients. This study revealed significant heterogeneity in both the clinical outcomes and CPET findings across these classifications [38]. Ho et al. used the ESC, American College of Cardiology/American Heart Association (ACC/AHA), and Heart Failure Society of America (HFSA) classifications among their patients with symptomatic HFpEF (NYHA II-IV) and found significant heterogeneity in both the clinical outcomes and CPET findings. This heterogeneity was attributed to the variety of comorbidities often present in HFpEF patients, such as diabetes, obesity, atrial fibrillation, and hypertension, which complicate their management and risk stratification. To address this complexity, right heart catheterization (RHC) plays a critical role in risk stratification for AHF patients, providing essential hemodynamic measurements that help to assess prognosis and predict outcomes [39]. A study conducted on 657 heart transplant recipients in Italy (2000–2018) identified a Pulmonary Artery Pulsatility Index (PAPI) value of <1.68 (low PAPI) as a predictor of post-transplant complications, including renal replacement therapy and primary graft dysfunction [40]. Analysis from the Evaluation Study of Congestive Heart Failure and Pulmonary Catheterization Effectiveness (ESCAPE) trial revealed that an elevated right atrial pressure to pulmonary capillary wedge pressure (RAP/PCWP) ratio was linked to adverse clinical outcomes in LVAD and transplant patients [41]. Pulmonary hypertension (PH) is a common finding among patients with AHF during RHC, and clinicians should differentiate it into three main groups: (1) isolated pre-capillary PH (PCWP ≤ 15 mmHg, mPAP > 20 mmHg, pulmonary vascular resistance (PVR) ≥ 3 Wood units (WUs)), (2) combined pre-/post-capillary PH (PCWP > 15 mmHg, mean pulmonary arterial pressure (mPAP) > 20 mmHg, PVR ≥ 2 WUs), and (3) isolated post-capillary PH (PCWP > 15 mmHg, mPAP > 20 mmHg, PVR < 2 WUs) [42]. This distinction is important because treatments designed for WHO group 1 pulmonary hypertension (PH) have been linked to worse outcomes in patients with AHF who have WHO group 2 PH. Cyrille-Superville et al. studied 846 HF patients from the PRognostic Evaluation During Invasive CaTheterization for Heart Failure (PREDICT-HF) registry who were invasively evaluated by RHC [43]. This study aimed to estimate survival to orthotopic heart transplant (OHT), durable LV assist device (LVAD), or death within six months as the primary endpoint. Twenty-one percent of all patients reached the primary endpoint, with Aortic Pulsatility Index (API) (OR 0.94; 95% CI: 0.91–0.96, *p* < 0.001) and Cardiac Power Output (CPO) (OR 0.76; 95% CI: 0.71–0.83, *p* < 0.001) showing strong associations with increased mortality rates. When used together, these measures provided additional prognostic value [43]. In addition to these markers, the Seattle Heart Failure Model (SHFM) is a validated tool that helps predict survival and inform treatment decisions for physicians [44]. It was derived from a cohort of 1125 HF patients using a multivariate Cox model and has been prospectively validated in five additional cohorts comprising 9942 patients and 17,307 person–years of follow-up. An overall receiver operating characteristic (ROC) area under the curve (AUC) of 0.729 further indicates its reliability and predictive strength. The SHFM also offers individualization options, as clinicians can modify medication parameters while using it so that they can optimize patient care [44]. Unlike other HF models, SHFM does not require invasive measures like peak VO_2_, which has been shown to contribute little predictive value. It outperformed other models, such as the Acute Decompensated Heart Failure National Registry (ADHERE) and the Heart Failure Survival Score (HFSS), particularly in patients on β-blockers. In addition to SHFM, the Interagency Registry for Mechanically Assisted Circulatory Support (INTERMACS) classification provides seven profiles to assess patients’ severity of heart failure: Profile 7 (Advanced NYHA Class III), Profile 6 (Exertion Limited), Profile 5 (Exertion Intolerant), Profile 4 (Resting Symptoms), Profile 3 (Stable, Inotrope Dependent), Profile 2 (Progressive Decline), and Profile 1 (Cardiogenic Shock) [45]. Samman-Tahhan et al. studied 969 patients with HF, 423 (43.7%) of whom were classified as stable Stage C patients because they were not matching any INTERMACS profile criteria [45]. The remaining patients were classified as follows: 348 (35.9%) in INTERMACS Profile 7, 146 (15.1%) in Profile 6, and 52 (5.4%) in Profiles 4 to 5. A comparison of the 3-year mortality rates revealed that mortality increased as the INTERMACS profiles were worsening. The 3-year mortality rate for stable Stage C (AHA) was 10%, while it was 21.8% (HR = 2.45, *p* < 0.001), 26% (HR = 3.93, *p* < 0.001), and 43.8% (HR = 6.35, *p* < 0.001) for patients in INTERMACS Profiles 7, 6, and 4 to 5, respectively. Similarly, the composite endpoint of death, LVAD, or heart transplantation had increasing rates from 11.3% in stable Stage C to 24.5% (HR = 2.49, *p* < 0.001), 31.6% (HR = 4.03, *p* < 0.001), and 51.3% (HR = 6.69, *p* < 0.001) in the INTERMACS 7, 6, and 4 to 5 groups, respectively. The hazard ratios for both mortality and the composite endpoint also increased, suggesting a higher risk of adverse outcomes. In general, patients with worse INTERMACS profiles had significantly higher mortality and event rates [45]. The INTERMACS profiles provided better prognostic differentiation than the NYHA functional class for both mortality and the composite endpoint.

Diastolic dysfunction leads to inadequate filling and elevated filling pressures. This dysfunction can progress to diastolic HF, characterized by symptoms such as shortness of breath, fatigue, and fluid retention, despite a preserved EF. Infiltrative cardiomyopathies, such as amyloidosis and sarcoidosis, are notable causes of diastolic dysfunction. These conditions involve the deposition of abnormal substances within the myocardial tissue, resulting in increased stiffness and impaired ventricular compliance [46]. Consequently, the ventricles struggle to fill properly during diastole, leading to elevated intracardiac pressures and heart failure symptoms.

Accurate diagnosis of diastolic dysfunction, particularly in the context of infiltrative diseases, is crucial for effective management. Traditionally, LVEDP measurement via cardiac catheterization has been the gold standard for assessing diastolic function. However, this invasive procedure carries certain risks. Recent studies have explored the utility of non-invasive biomarkers in predicting elevated LVEDP. For instance, research has demonstrated that biomarkers such as NT-proBNP and soluble ST2 (sST2) correlate with LVEDP, suggesting their potential role in diagnosing diastolic dysfunction [47]. Incorporating these biomarkers with echocardiographic parameters may enhance the accuracy of non-invasive assessments, facilitating the earlier detection and management of diastolic dysfunction in patients with infiltrative cardiomyopathies.

Etiological treatment in infiltrative diseases that can lead to advanced HF is critical for improving cardiac function and overall prognosis. Infiltrative cardiomyopathies, such as sarcoidosis, Fabry disease, and amyloidosis, cause progressive myocardial damage due to abnormal substance deposition, leading to diastolic dysfunction and, eventually, advanced HF [48,49]. Identifying and addressing the underlying cause are essential to slowing down disease progression and optimizing patient outcomes.

For cardiac sarcoidosis, immunosuppressive therapy, particularly corticosteroids, remains the mainstay of treatment [50]. High-dose prednisone is often initiated to reduce granulomatous inflammation and prevent conduction abnormalities and ventricular arrhythmias. In cases where steroids are insufficient or poorly tolerated, steroid-sparing agents like methotrexate, azathioprine, or mycophenolate mofetil are used [51]. Advanced cases with refractory ventricular arrhythmias or conduction disease may require implantable cardioverter-defibrillators (ICDs) or even mechanical circulatory support in end-stage HF.

In Fabry disease, enzyme replacement therapy (ERT) with recombinant α-galactosidase A (agalsidase alfa or beta) is the cornerstone of treatment, aiming to reduce glycosphingolipid accumulation and slow down disease progression [52]. More recently, chaperone therapy (migalastat) has been approved for patients with amenable mutations, offering an oral alternative to ERT [53]. In patients with significant left ventricular hypertrophy, additional HF therapies such as renin–angiotensin–aldosterone system inhibitors and beta-blockers may be used, although caution is needed due to conduction system disease.

For cardiac amyloidosis, treatment depends on the amyloid subtype. In light-chain (AL) amyloidosis, chemotherapy with agents such as bortezomib, cyclophosphamide, and dexamethasone is essential to reducing amyloid production [54]. In transthyretin (ATTR) amyloidosis, tafamidis is now the first-line therapy, stabilizing transthyretin to prevent further fibril deposition [55]. Other emerging agents, such as patisiran and inotersen (gene silencing therapies), target transthyretin synthesis and are promising for hereditary ATTR amyloidosis. Supportive therapy, including diuretics and anticoagulation, is essential in managing symptoms, though traditional HF medications such as beta-blockers and ACE inhibitors may be poorly tolerated in these patients.

Overall, the etiological treatment of infiltrative cardiomyopathies requires a disease-specific approach, often involving targeted therapies, immunosuppression, or enzyme replacement, alongside conventional HF management strategies. Early recognition and intervention are crucial in preventing irreversible cardiac damage and progression to advanced HF. 

## 3. Therapeutic Approaches in Advanced Heart Failure

AHF represents the end-stage of a progressive disease, whereby conventional therapies become inadequate to sustain quality of life or survival. The management of this condition requires a multidisciplinary approach, combining pharmacological interventions, device-based therapies, mechanical circulatory support, and, in some cases, heart transplantation. For patients who are ineligible for invasive procedures, palliative care plays a crucial role in symptom alleviation and quality of life enhancement.

### 3.1. Pharmacological Therapies

Intravenous inotropic agents, such as dobutamine and milrinone, are commonly initiated in the acute setting for hemodynamic stabilization and to improve end-organ perfusion [56]. These agents work by enhancing myocardial contractility and reducing systemic vascular resistance, thereby improving tissue perfusion. However, prolonged use of inotropes is associated with adverse effects, including arrhythmias, increased mortality, and dependence [57]. Therefore, they are generally reserved for bridging patients to definitive therapies, such as transplantation or mechanical circulatory support, or for palliative symptom management in end-stage disease. The decision to arrange for chronic continuous infusions after hospital discharge should be guided by the need for symptom relief and patient preferences [56]. The majority of patients on home inotropic infusions die by 6 months, and almost all are dead by 1 year, most often due to terminal hemodynamic decompensation [56]. Two big randomized controlled trials, the Randomized Evaluation of Mechanical Assistance for the Treatment of Congestive Heart Failure (REMATCH) [58] and Investigation of Nontransplant-Eligible Patients Who Are Inotrope-Dependent (INTrEPID) [59] trial, have shown superiority of mechanical circulatory support over inotropic therapy in patients with Stage D HF who require lifelong therapy.

Symptom management in AHF often involves addressing fluid retention, dyspnea, and fatigue. Diuretics remain the cornerstone for managing volume overload, but careful monitoring is necessary to prevent electrolyte imbalances and renal dysfunction [60]. Vasodilators, such as nitrates and hydralazine, are used to reduce afterload and improve cardiac performance, especially in patients with HFpEF [61,62]. Beta-blockers and RAAS inhibitors, though foundational in earlier stages, may require dose adjustments or discontinuation in advanced disease due to hemodynamic intolerance [4,63].

### 3.2. Device-Based Interventions

Secondary mitral regurgitation (SMR) is a common complication in AHF, contributing to worsening symptoms and progression of the disease [64]. The MitraClip device offers a minimally invasive solution to reduce SMR by clipping the mitral valve leaflets together, thereby improving valve coaptation [64]. Clinical trials, such as the Cardiovascular Outcomes Assessment of the MitraClip Percutaneous Therapy for Heart Failure Patients with Functional Mitral Regurgitation (COAPT) study [65], have demonstrated that transcatheter mitral valve repair leads to significant reductions in hospitalizations and lower all-cause mortality within 24 months of follow-up among patients with heart failure and moderate-to-severe or severe SMR who remained symptomatic despite the use of maximal doses of guideline-directed medical therapy. However, patient selection remains critical, as those with severe LV dysfunction may derive less benefit.

Implantable cardioverter-defibrillators (ICDs) play a crucial role in preventing sudden cardiac death in patients with AHF and reduced ejection fraction [60,66,67]. These devices deliver shocks or pacing to terminate life-threatening ventricular arrhythmias. Cardiac resynchronization therapy (CRT), often combined with ICDs, involves biventricular pacing to improve ventricular synchrony and cardiac output [68]. CRT is particularly beneficial in patients with left bundle branch block and significant QRS prolongation, leading to improved survival, reduced hospitalizations, and enhanced functional capacity [69,70].

Cardiac contractility modulation (CCM) is an innovative device-based therapy designed to enhance the strength of ventricular contractions in patients with heart failure, particularly those with reduced ejection fraction who are not candidates for cardiac resynchronization therapy. The CCM device delivers biphasic electrical signals to the ventricular myocardium during the absolute refractory period, a phase in which the heart muscle is unresponsive to new stimuli [71]. This non-excitatory stimulation does not initiate additional heartbeats but modulates myocardial contractility, leading to improved cardiac output.

Clinical studies have demonstrated that CCM therapy can lead to significant improvements in patients’ quality of life, functional status, and exercise capacity. Specifically, patients receiving CCM have shown enhancements in left ventricular ejection fraction, peak oxygen uptake, and reductions in heart failure-related hospitalizations [72]. At the cellular level, CCM promotes favorable remodeling by restoring calcium handling, improving metabolic efficiency, and reversing maladaptive gene expression associated with heart failure. These molecular changes contribute to the overall therapeutic benefits observed in patients undergoing CCM therapy [72].

The implantation of the CCM device involves a procedure similar to that of a pacemaker, where leads are placed in the right ventricular septum to deliver therapeutic signals. As of 2016, experienced centers have reported successful implantation practices, highlighting the safety and efficacy of the procedure. As the adoption of CCM therapy expands, ongoing research continues to explore its potential applications, including its role in patients with HFpEF, aiming to broaden the scope of individuals who may benefit from this therapy [73].

### 3.3. AF Ablation and Pacing Strategies in HF

Ablation and pacing strategy is a therapeutic approach for managing patients with AF and HF, particularly when conventional treatments are ineffective. This method involves performing atrioventricular node ablation (AVNA) to prevent irregular and rapid electrical impulses from reaching the ventricles, followed by the implantation of a permanent pacemaker to maintain a regular heart rhythm. Recent studies have explored the efficacy of conduction system pacing (CSP), such as left bundle branch pacing (LBBP), in conjunction with AVNA. The PACE-FIB trial, for instance, is a multicenter, prospective study comparing LBBP combined with AVNA to optimal pharmacological rate control in patients with HFpEF or HFmrEF and permanent AF. The primary outcome focuses on a composite of all-cause mortality, HF hospitalization, and worsening HF over 36 months [74].

Comparative analyses have also been conducted between different pacing modalities post-AVNA. A study by Ivanovski et al. evaluated clinical outcomes in HF patients with refractory AF who underwent either biventricular (BiV) pacing or CSP following AVNA. The findings indicated that CSP modalities, including His-bundle pacing (HBP) and LBBP, were associated with superior symptomatic and echocardiographic improvements compared to BiV pacing [75]. Notably, patients receiving CSP demonstrated significant enhancements in left ventricular ejection fraction and NYHA functional class. These results suggest that CSP, particularly LBBP due to its stable pacing parameters, may offer a more effective pacing strategy in the ablate and pace approach for this patient population [75].

### 3.4. Mechanical Circulatory Support

LVADs have revolutionized the management of AHF by providing mechanical support to maintain systemic perfusion. These devices are used either as a bridge-to-transplant (BTT) or as destination therapy (DT) for patients ineligible for transplantation [76]. Candidates for LVAD therapy typically have end-stage HF refractory to medical and device-based therapies, with persistent symptoms and evidence of end-organ dysfunction. Comprehensive assessment, including psychosocial evaluation, is essential to ensure adherence to the complex care regimen required for LVAD maintenance.

LVADs improve survival, functional status, and quality of life [77]. Studies report 1-year survival rates exceeding 80% with LVAD therapy, which are comparable to heart transplantation outcomes [78]. Quality of life improvements include enhanced exercise capacity and reduced hospitalizations [77]. However, they are not without complications. Common issues include device-related thromboembolic events, gastrointestinal bleeding, infections, and right HF [79]. Advances in technology, such as smaller, more durable devices with improved hemocompatibility, continue to expand the indications for LVAD use.

### 3.5. Heart Transplantation

Heart transplantation remains the gold standard therapeutic approach for patients with AHF, offering the potential for prolonged survival and restored quality of life. Transplant eligibility is determined by factors such as irreversible end-stage HF, absence of significant comorbidities, and adherence to medical management. Absolute contraindications include active malignancy, systemic infection, and severe PH [80,81]. Advances in immunosuppression and post-transplant care have significantly improved outcomes, with median survival exceeding 12 years in adult recipients [82,83]. Long-term complications, such as chronic allograft vasculopathy and malignancies related to immunosuppression, require ongoing surveillance and management [84].

### 3.6. Palliative Care

Palliative care strategies include optimizing medical therapy, providing psychosocial support, and facilitating hospice care when appropriate [85].

Palliative care interventions focus on alleviating symptoms such as dyspnea, pain, and fatigue while maintaining dignity and quality of life [86]. Advanced care planning, including discussions about goals of care and preferences for end-of-life treatment, is integral to this approach. Multidisciplinary teams, involving cardiologists, palliative care specialists, social workers, and chaplains, ensure comprehensive care tailored to the patient’s needs and values.

For patients who are ineligible for transplantation or LVADs, palliative care strategies include optimizing medical therapy, psychosocial support, and facilitating hospice care when appropriate [85,87]. The focus shifts from prolonging life to enhancing the quality of remaining life, addressing both physical and emotional suffering.

## 4. Future Perspectives

### 4.1. Novel Pharmacological and Device Therapies

Advances in the management of AHF are being actively explored through ongoing clinical trials regarding novel pharmacological and device-based therapies. A significant focus lies in novel pharmacological agents targeting unique pathways of myocardial injury, inflammation, and fibrosis. Agents like omecamtiv mecarbil, a myosin activator, are being studied to further refine outcomes in AHF populations [88]. Specifically, the Acute Treatment with Omecamtiv Mecarbil to Increase Contractility in Acute Heart Failure (ATOMIC-HF) trial is a phase IIb clinical study that follows a double-blind, randomized, and placebo-controlled design with sequential cohorts. Its objective is to assess the effectiveness of an intravenous formulation of omecamtiv mecarbil in a population of around 600 patients who have been hospitalized due to acute decompensated HF. The trial is structured into three consecutive groups, each receiving progressively higher doses of the treatment [89]. The primary endpoint of this study was the effect on dyspnea within 48 h of intravenous mecamtiv mecarbil administration, while the secondary and exploratory objectives were focused on assessing safety, tolerability, pharmacokinetics, and echocardiographic markers. Upon trial completion, the results indicated that intravenous omecamtiv mecarbil did not lead to a notable improvement in dyspnea symptoms among patients hospitalized with acute decompensated HF. The Chronic Oral Study of Myosin Activation to Increase Contractility in Heart Failure (COSMIC-HF) trial is a phase II, multicenter, randomized, double-blind, placebo-controlled study aiming to thoroughly evaluate the effects of an orally administered, modified, sustained-release formulation of omecamtiv mecarbil in patients suffering from HF with LV systolic dysfunction [90]. The primary endpoint was to evaluate the peak plasma concentration of omecamtiv mecarbil. Secondary endpoints included assessing changes from the baseline in key clinical parameters at week 20, such as systolic ejection time, stroke volume, LV end-systolic and end-diastolic diameters, heart rate, and NT-proBNP levels. Additionally, the safety and tolerability of omecamtiv mecarbil were evaluated by monitoring the incidence of adverse events from the baseline to week 24. The trial showed that the use of omecamtiv mecarbil was associated with significant improvements in cardiac function and a reduction in ventricular dimensions, without superiority in adverse and clinical events compared to placebo. The Lowering Adverse Cardiac Outcomes Through Improving Contractility in Heart Failure (GALACTIC-HF) trial, a phase III, double-blind, placebo-controlled, randomized trial, primarily assessed time to cardiovascular (CV) or first HF event after the administration of omecamtiv mecarbil, while the secondary outcomes included the duration until CV death, patient-reported health status evaluated through the Kansas City Cardiomyopathy Questionnaire, time until the first hospitalization due to HF, and overall survival time measured by time to all-cause death [91]. The authors found that omecamtiv mecarbil successfully achieved the primary composite efficacy endpoint, resulting in a statistically significant reduction in CV mortality or HF-related events. Additionally, patients receiving omecamtiv mecarbil experienced fewer symptoms and lower NT-proBNP levels compared to those on placebo. Finally, the last trial on omecamtiv mecarbil was a double-blind, placebo-controlled, randomized trial, the Multicenter Exercise Tolerance Evaluation of Omecamtiv Mecarbil Related to Increased Contractility in Heart Failure (METEORIC-HF), evaluating changes in peak oxygen consumption during cardiopulmonary exercise testing, along with variations in peak exercise capacity, ventilatory efficiency, and average daily activity levels in patients with HFrEF [92]. Interestingly, omecamtiv mecarbil failed to show statistically significant alterations in peak VO_2_, maximal exercise workload, and ventilatory efficiency compared to the placebo group.

Novel SGLT2 inhibitors including dapagliflozin, empagliflozin, and sotagliflozin have been widely studied in both HFpEF and HFrEF. Characteristically, the Dapagliflozin in Patients with Heart Failure and Reduced Ejection Fraction (DAPA-HF) [93] and the Empagliflozin Outcome Trial in Patients with Chronic Heart Failure with Reduced Ejection Fraction (EMPEROR-Reduced) [94] trials showed significant reductions in the primary composite outcome and total HF hospitalizations in HFrEF patients after therapy with dapagliflozin and empagliflozin, respectively. In HFpEF patients, the Empagliflozin in Heart Failure with a Preserved Ejection Fraction (EMPEROR-Preserved) trial [95] demonstrated a significant reduction in the primary composite outcome and in total HF hospitalizations in the empagliflozin group compared to the controls, while in the Dapagliflozin Evaluation to Improve the Lives of Patients with Preserved Ejection Fraction Heart Failure (DELIVER) [96] trial, a significant reduction in the primary composite outcome and in worsening HF events in the dapagliflozin group was observed. However, there were more randomized trials in worsening HF, such as the Effect of Sotagliflozin on Cardiovascular Events in Patients with Type 2 Diabetes Post Worsening Heart Failure (SOLOIST-WHF) [97] and the Empagliflozin in Patients Hospitalized for Acute Heart Failure who have been Stabilized (EMPULSE) trials [98]. The first trial demonstrated a statistically significant reduction in primary endpoint events following sotagliflozin administration in patients hospitalized for worsening HF. The second trial found that empagliflozin was superior in 53.9% of paired comparisons for the composite outcome, which included time to all-cause death, number of HF exacerbations, time to first exacerbation, and a ≥5-point improvement in KCCQ-TSS after 90 days of treatment. In contrast, placebo was superior in 39.7% of comparisons, while 6.4% resulted in ties. Concurrently, device-based interventions, such as transcatheter mitral and tricuspid valve therapies, are being investigated to optimize cardiac function and reduce symptoms in patients’ refractory to medical therapy. Two recent randomized controlled trials compared MitraClip to medical therapy in patients with secondary MR due to left ventricular (LV) dysfunction. Specifically, the COAPT trial compared MitraClip therapy versus GDMT in 614 patients with severe secondary MR and LV dysfunction, showing a reduced hospitalization rate due to HF after 24 months (35.8%) compared to the medical therapy group (67.9%) and lower all-cause mortality compared to medical therapy alone (29.1% vs. 46.1%) [65]. On the contrary, the Multicenter Investigation of Transcatheter Mitral Valve Repair-France (MITRA-FR) trial showed no difference between both groups regarding mortality rate (24.3% in the MitraClip group vs. 22.4% in the medical group) and hospitalizations due to HF worsening (48.7% in the MitraClip group vs. 47.4% in the medical group) in 304 symptomatic patients with SMR and LV dysfunction [99]. Newer promising transcatheter mitral valve replacement prostheses include Tendyne (Abbott Vascular, Abbott Park, IL, USA), Intrepid (Medtronic, Minneapolis, MN, USA), Tiara (Neovasc Inc., Richmond, BC, Canada), CardiaQ (Edwards Lifesciences Corp., Irvine, CA, USA), Caisson (LivaNova PLC, London, UK), Cardiovalve (Cardiovalve, Yehuda, Israel), M3/Sapien (Edwards Lifesciences Corp., Irvine, CA, USA), and Highlife (Highlife SAS, Paris, France) [100].

Given the high mortality of tricuspid valve (TV) surgery, multiple transcatheter therapies for tricuspid intervention are being investigated, including edge-to-edge repair, TV replacement, tricuspid annuloplasty, and palliative tricuspid therapy. Edge-to-edge tricuspid valve repair includes the TriClip device with the ongoing Trial to Understand the Impact of the MitraClip Intervention in Subjects with Tricuspid Regurgitation TRILUMINATE Pivotal trial [101] and PASCAL transcatheter valve repair system with the CLASP TR early feasibility study [102]. Tricuspid valve replacement includes the EVOQUE tricuspid valve replacement system with the ongoing Trial of the Intecardia Endospacer System for Tricuspid Regurgitation (TRISCEND II) pivotal trial defij [103], the GATE system [104], the INTREPID system with the ongoing Transcathether Tricuspid Valve Replacement (TTVR) early feasibility study [105], and the LuX-Valve [106]. Tricuspid annuloplasty includes the TriCinch™ (4Tech Cardio Ltd., Galway, Ireland) with the Percutaneous Treatment of Tricuspid Valve Regurgitation With the TriCinch™ System (PREVENT) trial [107], the Trialign™ device (Mitralign, Inc., Boston, MA, USA) with the ongoing Safety and Performance of the Trialign Percutaneous Tricuspid Valve Annuloplasty System for Symptomatic Chronic Functional Tricuspid Regurgitation (SCOUT II) trial [108], and the pledget-assisted suture tricuspid annuloplasty (PASTA) technique [109].

The development of next-generation LVADs is another critical area of exploration. Efforts to miniaturize devices, improve hemocompatibility, and extend battery life aim to reduce complications like pump thrombosis and infection. Fully implantable LVADs, which eliminate the need for percutaneous driveline connections, are undergoing evaluation to enhance patient mobility and quality of life [110,111]. Moreover, new sensors integrated into LVAD systems are being tested to enable the real-time monitoring of hemodynamic parameters, potentially allowing for the earlier detection of device-related complications [111]. A notable example is the PReduction pump (Pumpinheart Limited, Dublin, Ireland), a long-lasting partial support device implanted through a transcatheter method across the mitral valve, serving as a diastolic assist system [112]. The need for durable mechanical circulatory support (MCS) devices designed for smaller patients, such as children with AHF and women, remains a major clinical challenge. A key example is Abbott, Inc., which is creating the HeartMate 3 Mini, a more compact version of the HeartMate 3 [111].

### 4.2. AI and Machine Learning in HF Management

Innovative technologies are a crucial component in the treatment and management of AHF. Artificial intelligence (AI) and machine learning (ML) are revolutionizing HF management by enabling predictive modeling, early detection, and personalized therapeutic strategies. Advanced algorithms are being developed to analyze complex datasets, including electronic health records, imaging, and biomarker profiles, to predict decompensation events and guide interventions [113,114]. AI-driven decision support tools are also being integrated into clinical workflows to enhance diagnostic accuracy and optimize treatment plans for patients with AHF.

### 4.3. Advances in Remote Monitoring and Wearable Devices

Remote monitoring technologies, including wearable devices, are gaining traction for their ability to continuously track vital signs and physiological parameters. Innovations such as implantable hemodynamic monitors and smart patches equipped with biosensors are being developed to provide actionable insights in real time [115,116]. These technologies not only facilitate early intervention in response to signs of worsening HF but also empower patients to actively engage in their own care. Future iterations of wearable devices are expected to incorporate AI capabilities for enhanced data interpretation and individualized feedback.

### 4.4. Personalized Medicine

Personalized medicine is emerging as a cornerstone of AHF management. Genomic research has identified numerous genetic variants associated with HF susceptibility and progression, paving the way for genotype-based therapeutic strategies [117,118,119]. Biomarkers such as NT-proBNP, galectin-3, and soluble ST2 are being further investigated to stratify risk and tailor treatment in AHF populations [120,121]. Additionally, individualized approaches, including pharmacogenomics and tailored device programming, are being explored to maximize therapeutic efficacy while minimizing adverse effects. Precision phenotyping using multimodal data integration is anticipated to further refine these individualized strategies [122].

### 4.5. Preventive Strategies

The prevention of AHF decompensation is increasingly focused on the integration of multidisciplinary care models. These programs bring together cardiologists, HF specialists, nurses, and allied health professionals to provide holistic, patient-centered care. Enhanced outpatient HF programs emphasizing education, dietary counseling, and symptom monitoring are showing promise in reducing hospital readmissions and improving quality of life [123]. Future preventive strategies are likely to incorporate digital health tools and telemedicine to extend the reach and efficiency of these interventions.

## 5. Limitations in Advanced Heart Failure

Although there are promising future perspectives in the field of AHF, significant limitations remain due to gaps in the literature. Identifying patients at risk of transitioning to AHF remains a significant challenge. Current diagnostic tools, including imaging and biomarkers, may lack the sensitivity to detect subclinical disease progression [124]. The heterogeneity of HF presentations further complicates timely recognition, delaying the initiation of appropriate interventions.

Access to advanced therapies such as LVADs, heart transplantation, and novel pharmacological agents is often limited by resource constraints, geographic disparities, and financial barriers [125]. These limitations disproportionately affect underserved populations, exacerbating healthcare inequities and contributing to suboptimal outcomes in AHF management.

Despite the promise of AI, remote monitoring, and wearable devices, their integration into routine clinical practice is still limited. Challenges include technological complexity, data management issues, and the need for clinician training. Furthermore, regulatory and reimbursement frameworks have yet to fully adapt to the rapid pace of innovation, slowing down the widespread adoption of these tools [126,127].

Moreover, while personalized medicine holds great potential, its application is hindered by the lack of standardization in genetic testing and biomarker validation. Moreover, integrating genomic and phenotypic data into clinical workflows requires sophisticated infrastructure and expertise, which are not universally available [128]. Ethical considerations related to genetic data privacy and patient consent also pose challenges.

Finally, the successful implementation of preventive strategies relies on multidisciplinary collaboration and patient adherence. However, systemic barriers such as workforce shortages, fragmented care delivery, and patient-level factors like health literacy and socioeconomic status can limit the effectiveness of these programs [129]. Sustaining long-term engagement in preventive initiatives remains an ongoing challenge.

## 6. Conclusions

In conclusion, the transition to AHF marks a pivotal phase in the continuum of CV disease, necessitating early identification and proactive management to improve patient outcomes. Timely recognition of clinical and hemodynamic indicators of disease progression enables the initiation of advanced therapies, including pharmacological optimization, device-based interventions, and candidacy for transplant or mechanical circulatory support. Collaborative, multidisciplinary care plays a crucial role in tailoring treatment strategies to individual patient needs, emphasizing both symptom relief and quality of life. By integrating innovative technologies, personalized medicine, and robust palliative care, the approach to managing AHF continues to evolve, offering hope for prolonged survival and improved prognosis in this challenging patient population.

## Figures and Tables

**Table 1:** Epidemiological characteristics of AHF.

Parameter	Value/Percentage
Percentage of AHF among patients with HF	5–10% [10,15]
Annual mortality rate	20–50% [16]
Hospitalizations	80% of AHF patients are hospitalized per year [16]
Incidence in patients > 65 years old	10% [10]
Diabetes mellitus	40% [16]
Chronic kidney disease	30–50% [16]

**Table 2:** Gender and racial disparities in AHF.

Parameter	Male	Female	African American	Caucasian
Prevalence	Higher	Lower	Double	Lower [15]
Comorbidities	Ischemic HF	Hypertension	Hypertension	Diabetes [15,16]
Mortality rate	40–50%	30–40%	Higher	Lower [16]

## Data Availability

Not applicable.
